# Innate immune activity as a predictor of persistent insulin secretion and association with responsiveness to CTLA4-Ig treatment in recent-onset type 1 diabetes

**DOI:** 10.1007/s00125-018-4708-x

**Published:** 2018-08-30

**Authors:** Susanne M. Cabrera, Samuel Engle, Mary Kaldunski, Shuang Jia, Rhonda Geoffrey, Pippa Simpson, Aniko Szabo, Cate Speake, Carla J. Greenbaum, Type 1 Diabetes TrialNet CTLA4-Ig (Abatacept) Study Group, Yi-Guang Chen, Martin J. Hessner

**Affiliations:** 1 Max McGee Research Center for Juvenile Diabetes, Children’s Research Institute of Children’s Hospital of Wisconsin, Milwaukee, WI USA; 2 Department of Pediatrics, Section of Endocrinology, Medical College of Wisconsin, 8701 Watertown Plank Road, Milwaukee, WI 53226 USA; 3 Department of Pediatrics, Division of Quantitative Health Sciences, Medical College of Wisconsin, Milwaukee, WI USA; 4 Division of Biostatistics, Medical College of Wisconsin, Milwaukee, WI USA; 5 Diabetes Clinical Research Program, Benaroya Research Institute, Seattle, WA USA

**Keywords:** Biomarker, CTLA4-Ig, Disease heterogeneity, Honeymoon, Partial remission, Therapeutic response, Type 1 diabetes

## Abstract

**Aims/hypothesis:**

The study aimed to determine whether discrete subtypes of type 1 diabetes exist, based on immunoregulatory profiles at clinical onset, as this has significant implications for disease treatment and prevention as well as the design and analysis of clinical trials.

**Methods:**

Using a plasma-based transcriptional bioassay and a gene-ontology-based scoring algorithm, we examined local participants from the Children’s Hospital of Wisconsin and conducted an ancillary analysis of TrialNet CTLA4-Ig trial (TN-09) participants.

**Results:**

The inflammatory/regulatory balance measured during the post-onset period was highly variable. Notably, a significant inverse relationship was identified between baseline innate inflammatory activity and stimulated C-peptide AUC measured at 3, 6, 12, 18 and 24 months post onset among placebo-treated individuals (*p ≤* 0.015). Further, duration of persistent insulin secretion was negatively related to baseline inflammation (*p* ≤ 0.012) and positively associated with baseline abundance of circulating activated regulatory T cells (CD4^+^/CD45RA^−^/FOXP3^high^; *p* = 0.016). Based on these findings, data from participants treated with CTLA4-Ig were stratified by inflammatory activity at onset; in this way, we identified pathways and transcripts consistent with inhibition of T cell activation and enhanced immunoregulation. Variance among baseline plasma-induced signatures of TN-09 participants was further examined with weighted gene co-expression network analysis and related to clinical metrics. Four age-independent subgroups were identified that differed in terms of baseline innate inflammatory/regulatory bias, rate of C-peptide decline and response to CTLA4-Ig treatment.

**Conclusions/interpretation:**

These data support the existence of multiple type 1 diabetes subtypes characterised by varying levels of baseline innate inflammation that are associated with the rate of C-peptide decline.

**Data availability:**

Gene expression data files are publicly available through the National Center for Biotechnology Information Gene Expression Omnibus (accession number GSE102234).

**Electronic supplementary material:**

The online version of this article (10.1007/s00125-018-4708-x) contains peer-reviewed but unedited supplementary material, which is available to authorised users.

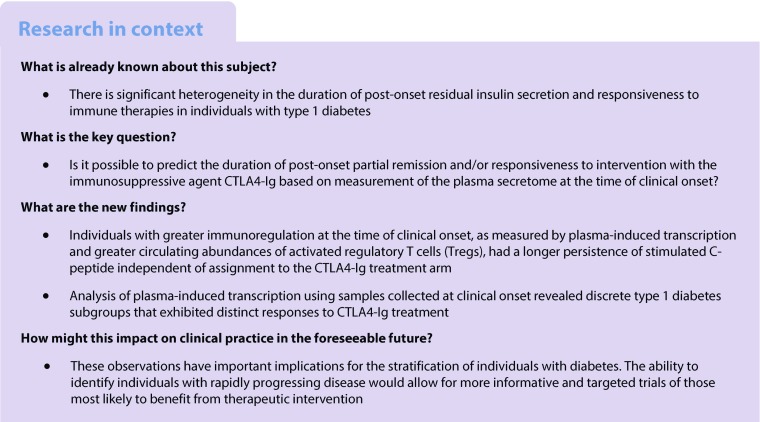



## Introduction

Type 1 diabetes arises through autoimmunity towards the pancreatic beta cells. Common features include the development of islet-antigen-specific autoantibodies and autoreactive T cells, as well as lifelong dependence on insulin-replacement therapy [1–4].

Numerous environmental factors and >40 genetic loci have been associated with susceptibility to type 1 diabetes [5, 6]. Varied combinations of these contributors likely underlie the significant disease heterogeneity observed among individuals. For example, while many individuals are diagnosed during childhood or adolescence, onset can occur at any age [7, 8]. The time between the presence of autoantibodies and clinical onset is also variable, but it is generally shorter in younger children [9]. Most individuals have residual endogenous insulin secretion at clinical diagnosis. Yet the amount of insulin secretion, measured by mixed meal tolerance test (MMTT)-stimulated C-peptide, diminishes over time and, as during the preclinical period, the rate of fall is variable, but more rapid in younger children. Trials of disease-modifying therapy aim to preserve endogenous insulin secretion after clinical diagnosis, as persistence of C-peptide, particularly MMTT-stimulated values ≥0.2 nmol/l, are associated with a decreased risk of severe hypoglycaemia and microvascular complications [10–14].

In trials of disease-modifying immunotherapy, heterogeneity in the rate of disease progression poses challenges in detecting the effect of treatment on preservation of stimulated C-peptide. As many as 17% of individuals have no fall in C-peptide during the first year after diagnosis and thus would not benefit from an intervention [15]. In contrast, 12% of individuals have levels of stimulated C-peptide that rapidly decline to less than 0.2 nmol/l during the same time frame [15]. Measures capable of predicting the post-onset disease course and/or forecasting the response to therapeutic intervention would enable individual stratification and development of individualised therapies [16].

We are able to differentiate immune states with a sensitive bioassay whereby an individual’s plasma or serum is used to induce transcription in a well-controlled reporter cell population [17–20]. After co-culture, the response is measured with a microarray then subjected to ontological analysis and follow-up studies. We have reported a disease-specific, partially IL-1-dependent, transcriptional signature associated with diabetes progression that is detectable prior to the development of autoantibodies [21]. We have also identified a familial innate inflammatory state in healthy siblings of people with diabetes that is independent of HLA, autoantibody status and disease progression [22]. Among sibling non-progressors with high-risk HLA haplotypes, this state is temporally supplanted by an IL-10/TGF-β-mediated regulatory state associated with peripheral increases in activated (CD4^+^/CD45RA^−^/forkhead box P3 [FOXP3]^high^) regulatory T cell (Treg) frequencies. These observations suggest that diabetes may develop because of failures in endogenous regulatory mechanisms that normally manage underlying risk.

We previously applied our bioassay to the Anti-Interleukin-1 in Diabetes Action (AIDA) and TrialNet Canakinumab (TN-14) trials [23, 24]. While these trials failed to show overall efficacy of IL-1 receptor antagonist (IL-1Ra) or canakinumab, plasma-induced transcription suggested that both therapies achieved varying degrees of immunomodulation consistent with IL-1 inhibition. At the endpoint, we quantitatively scored the signatures and identified an inverse relationship between measured inflammation and stimulated C-peptide response in treated participants.

Here, we examine post-onset diabetes progression in local patients from the Children’s Hospital of Wisconsin and participants in the TrialNet CTLA4-Ig (Abatacept) trial (TN-09) [25]. The cytotoxic T lymphocyte-associated protein 4/Fc fraction of the IgG_1_ fusion protein (CTLA4-Ig), also known as abatacept, inhibits CD28-mediated T cell co-stimulation by binding to CD80/86 on antigen-presenting cells. In TN-09, CTLA4-Ig slowed the decline of beta cell function in treated participants—an effect possibly mediated through modulation of central memory CD4 T cells [26]. Using plasma-induced transcription and a refined scoring algorithm, we investigated how heterogeneity at clinical diagnosis relates to persistence of insulin secretion during the post-onset period and responsiveness to CTLA4-Ig treatment.

## Methods

### Study participants

Local participants (*n* = 42) were recruited through the Children’s Hospital of Wisconsin (Milwaukee, WI, USA) and samples were collected as described previously [22]. Institutional review board approval (CHW IRB 01–15) was granted for all analyses, and informed consent/assent was obtained for all participants. In TN-09 (NCT00505375), participants were randomised in a 2:1 ratio to receive CTLA4-Ig (abatacept) (10 mg/kg, maximum 1000 mg/dose) or placebo (saline [154 mmol/l NaCl]) intravenously on days 1, 14 and 28 and monthly for a total of 27 infusions over 2 years [25]. At baseline, participants were <100 days post-diagnosis and had MMTT-stimulated C-peptide ≥0.2 nmol/l within 21 days of diagnosis. Based on availability, we procured serum collected at 0 (baseline), 3, 12 and 24 months from 74 of the 112 TN-09 participants (66.1%) for this ancillary analysis. The 54 CTLA4-Ig-treated participants analysed exhibited a higher mean baseline-adjusted 2 h AUC stimulated C-peptide response at the 24 month endpoint (0.49 ± 0.54 nmol/l) compared with the 20 placebo-treated participants (0.29 ± 0.25 nmol/l); this difference did not reach significance with the number of participants tested (*p* = 0.11). Relative to other metrics (electronic supplementary material [ESM] Table 1), this subset was not different compared with the complete TN-09 cohort. Compared with the local cohort, this subset exhibited an older age at onset (14.7 ± 6.8 vs 9.7 ± 3.3 years) and a shorter duration of diabetes at the baseline visit (0.24 ± 0.04 years vs 0.30 ± 0.14 years).

### Preservation of insulin secretion

In the TN-09 participants, preservation of insulin secretion was defined as the duration of time that MMTT-stimulated C-peptide remained ≥0.2 nmol/l. As MMTTs were not performed in the local cohort, the duration of time that the insulin-dose-adjusted A_1c_ (IDAA1c) remained ≤9 was used as a surrogate definition [27].

### Plasma-induced transcription

Cryopreserved peripheral blood mononuclear cells (PBMCs) from a single healthy blood donor (Cellular Technology, Shaker Heights, OH, USA) were cultured in 300 μl RPMI 1640 medium with 200 μl plasma (local participants) or serum (TN-09 participants) and induced transcription was measured using Affymetrix GeneChip Human Genome U133 plus 2.0 arrays (Affymetrix, Santa Clara, CA, USA), as described previously [18, 22, 24].

### Data analysis

All array data were subjected to global median normalisation with Bioconductor robust multi-array analysis [28]. The significance of differentially induced transcription was assessed through ANOVA and the rate of type I errors in multiple testing was assessed from false-discovery rates (FDRs) determined with Partek Genomics Suite 6.6 (Partek, Saint Louis, MO, USA). To optimise scoring of plasma-induced transcription, probe sets that were the best classifiers of new-onset diabetes were identified by re-analysing prior cross-sectional data [22] with random forest analysis (Salford Systems, San Diego, CA, USA), a tree-based ensemble machine learning tool. Ontological analysis used Ingenuity Pathway Analysis (IPA, QIAGEN, Redwood City, CA, USA). Hierarchical clustering was conducted with Genesis [29]. Relationships between the induced gene expression signatures and clinical phenotypes were identified with weighted gene co-expression network analysis (WGCNA) [30].

### The ex vivo vs in vitro effect of CTLA4-Ig

PBMCs were pre-treated in medium with CTLA4-Ig (Bristol-Myers Squibb, New York, NY, USA) at 0 μg/ml, 25 μg/ml and 82 μg/ml for 45 min prior to the addition of plasma from a recently diagnosed local individual. Transcriptional analysis was conducted as detailed above.

### Flow cytometry

PBMCs were stained with the fixable LIVE/DEAD Violet dye (Invitrogen, Carlsbad, CA, USA) for 30 min on ice, followed by blocking of Fc receptors and staining for anti-CD4 (clone RPA-T4), anti-CD25 (clone 2A3), anti-CD45RO (clone UCHL1), anti-CD45RA (clone HI100) and anti-CD127 (clone HIL-7R-M21, BD Bioscience, San Jose, CA, USA) on ice for 30 min, then intracellular staining with anti-FOXP3 (clone PCH101, Affymetrix eBioscience, San Diego, CA, USA). Analysis was conducted with an LSR II flow cytometer (BD Bioscience).

## Results

### Refined measurement of immunological balance

Previously, we conducted cross-sectional plasma-induced transcriptional analyses of individuals recently diagnosed with type 1 diabetes, unrelated healthy control individuals lacking family history of autoimmunity and healthy autoantibody-negative siblings of individuals with type 1 diabetes possessing high-risk (DR3 and/or DR4) or low-risk (non-DR3/non-DR4) HLA haplotypes [22]. Among these cohorts we identified 1374 differentially induced probe sets. To quantitatively measure immune activity, we developed a gene-ontology-based composite inflammatory index (*I*.*I*._*com*_), calculated by determining the ratio of the mean intensity of the induced inflammatory genes to the mean intensity of the induced regulatory genes [22]. High scores reflect greater inflammatory bias and low scores reflect greater regulatory bias.

**Fig. 1:**
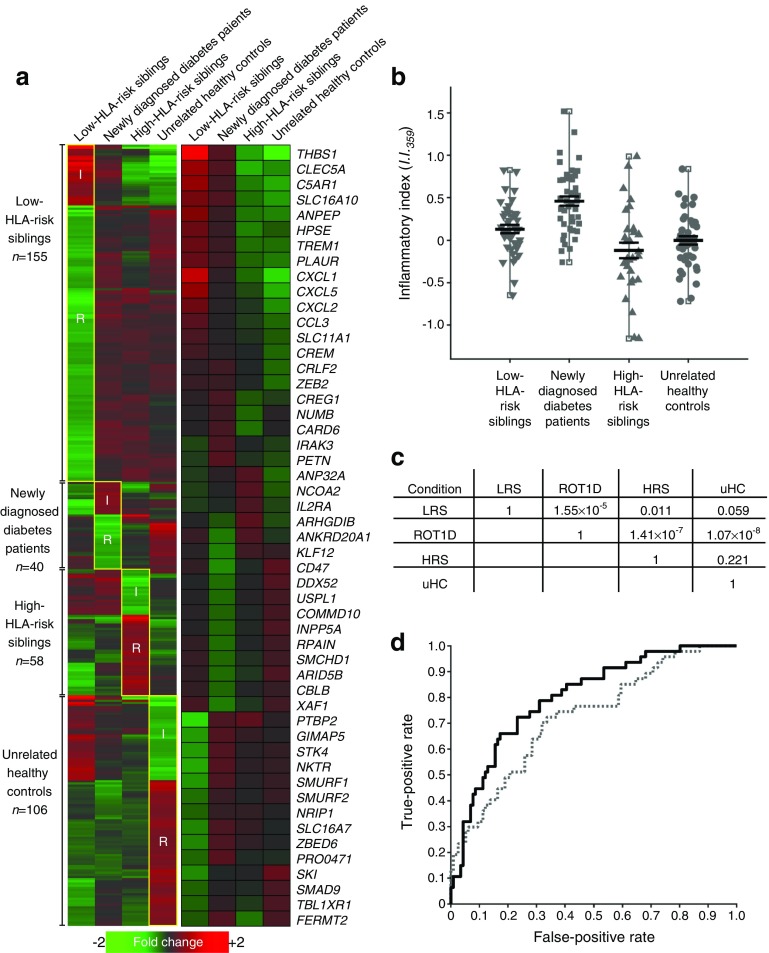
Refined scoring of plasma-induced transcriptional signatures. (**a**) Mean expression levels of the 359 probe sets that best distinguish individuals newly diagnosed with type 1 diabetes from the low-HLA-risk sibling, high-HLA-risk sibling, and unrelated healthy control cohorts. The analysis included: individuals with diabetes (*n* = 47, age 10.0 ± 2.9 years, blood glucose 8.6 ± 4.1 mmol/l, HbA_1c_ 5.8 ± 9.3 mmol/mol [7.5 ± 1.2%]; baseline samples were collected 2–7 months after diagnosis when metabolic control had been achieved); low-HLA-risk siblings (*n* = 42, age 8.4 ± 2.0 years, blood glucose 5.2 ± 0.9 mmol/l); high-HLA-risk siblings (*n* = 30, age 8.6 ± 1.9 years, blood glucose 5.2 ± 0.7 mmol/l); and unrelated healthy control individuals (*n* = 44, age 15.0 ± 4.1 years, blood glucose 5.1 ± 0.7 mmol/l). Random forest analysis used 12,589 probe sets identified in the six possible comparisons between the four cohorts at a fold change >1.1 and an FDR <20%. The probe sets that exhibited a random forest Gini score >3.49 when any one group was compared with the three others were retained. A second analysis identified probe sets that were regulated at log_2_ ratio >|0.263| (1.2-fold) and FDR <20% when one cohort was compared with any of the three others. A total of 359 transcripts met both criteria. (**a**) The left section shows the relative expression levels of the 359 probe sets across the four cohorts. The second analysis enabled the definition of four data subsets, the number of transcripts within each data subset is shown on the left. The transcripts generally annotated as inflammatory and regulatory within each data subset are indicated; the upregulated transcripts in the low-HLA-risk sibling and diabetic individual data subsets are generally annotated as inflammatory, while upregulated transcripts in the high-HLA-risk sibling and unrelated healthy control data subsets are generally annotated as regulatory. The annotated dataset is available from the corresponding author on request. The right section shows the mean expression levels of a subset of well-annotated transcripts. (**b**) Ontology-based scoring of cross-sectional samples using *I*.*I*._*359*_ significantly discriminates the diabetic cohort from the other cohorts. The mean *I*.*I*._*359*_ for the 47 cross-sectional diabetes participants (mean ± SE 0.46 ± 0.05) was significantly higher than that observed for the 42 siblings with low HLA risk (0.13 ± 0.05), 30 siblings with high HLA risk (−0.12 ± 0.09) and 44 unrelated healthy control participants (0.00 ± 0.05). *p* values are shown in panel (**c**); two-tailed unpaired *t* tests for the comparisons between each cohort. (**d**) ROC curve for 359 probe sets (solid line; AUC = 0.80) shows improved discrimination of the diabetic cohort from the related and unrelated control cohorts compared with the previously reported 1374 probe sets described in Chen et al [22] (dotted line; AUC = 0.72). ROT1D, recent-onset type 1 diabetes; LRS, low-HLA-risk sibling; HRS, high-HLA-risk sibling; I, inflammatory; R, regulatory; uHC, unrelated healthy control group

Here, in an effort to simplify the *I*.*I*._*com*_, we used random forest analysis to identify 359 probe sets that were optimal classifiers of the diabetic cohort relative to the three control cohorts. Among these 359 probe sets, 325 (90.5%) overlapped with the 1374 probe sets identified previously [22]. Similar to the original data, this subset captured increasing IL-10/TGF-β bias and decreasing IL-1/NFκB bias across the continuum created by the siblings with low HLA risk, individuals with type 1 diabetes, siblings with high HLA risk and unrelated control individuals (Fig. 1a). This is reflected by the increased induction of chemokine transcripts by plasma of siblings with low HLA risk and individuals with diabetes, and elevated induction of regulatory transcripts (*IL2RA*, *CBLB*, *SMURF1*, *SMURF2*, *SKI*) by plasma of siblings with high HLA risk and unrelated control individuals. As described for *I*.*I*._*com*_ [22], an inflammatory index based on these 359 probe sets (*I*.*I*._*359*_) was calculated by dividing the average signal intensity of the 103 probe sets generally annotated as ‘inflammatory’ by the average signal intensity of the 256 probe sets generally annotated as ‘regulatory’. Similar to *I*.*I*._*com*_, the average *I*.*I*._*359*_ for the individuals with diabetes was significantly higher than the other cohorts (Fig. 1b, c). The receiver operator characteristic (ROC) analysis of this subset showed improved discrimination of the diabetic and control cohorts (Fig. 1d).

### The relationship between baseline *I.I.*_*359*_ and beta cell function

**Fig. 2:**
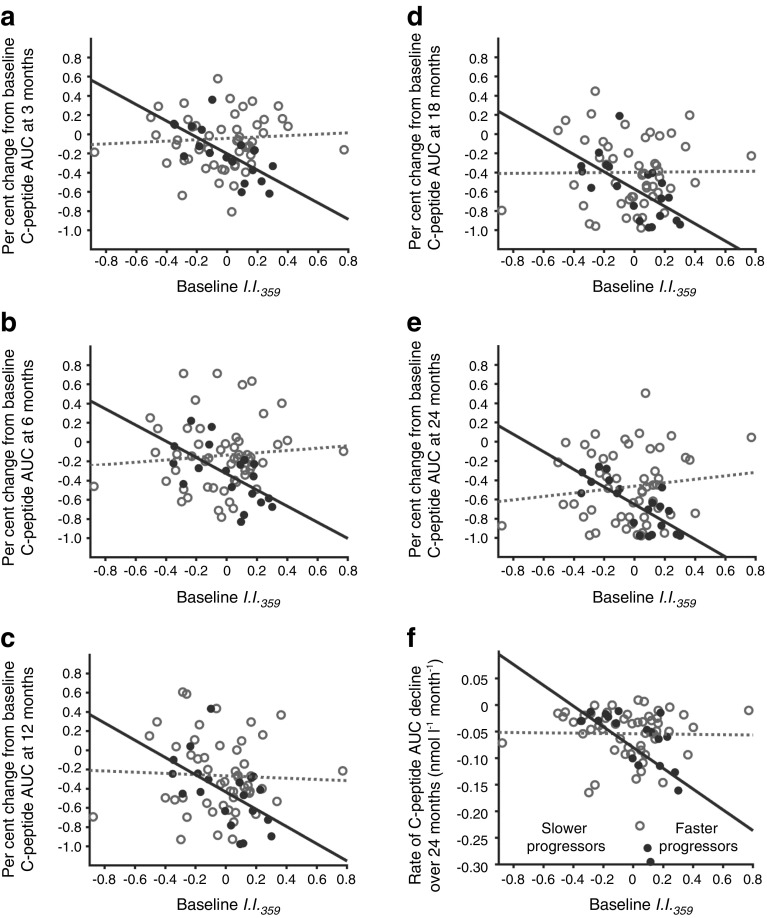
The relationship between baseline *I*.*I*._*359*_ and future beta cell function. A significant inverse relationship exists between baseline *I*.*I*._*359*_ and 2 h C-peptide AUC percent change from baseline at 3, 6, 12, 18 and 24 months in 19 placebo-treated TN-09 participants (all *p* < 0.015) but not in 54 CTLA4-Ig-treated TN-09 participants. (**a**) 3 months: placebo treated, slope = −0.85, *r*^2^ = 0.48, *p* = 0.001; CTLA4-Ig treated, slope = 0.07, *r*^2^ = 0.002, *p* = 0.731. (**b**) 6 months: placebo treated, slope = −0.84, *r*^2^ = 0.36, *p* = 0.006; CTLA4-Ig treated, slope = 0.12, *r*^2^ = 0.008, *p* = 0.522. (**c**) 12 months: placebo treated, slope = −0.90; *r*^2^ = 0.29; *p* = 0.015; CTLA4-Ig treated, slope = −0.06; *r*^2^ = 0.002; *p* = 0.768. (**d**) 18 months: placebo treated, slope = −0.90, *r*^2^ = 0.37; *p* = 0.004; CTLA4-Ig treated, slope = 0.01, *r*^2^ = 0.0001, *p* = 0.939. (**e**) 24 months: placebo treated, slope = −0.91, *r*^2^ = 0.56; *p* = 0.0002; CTLA4-Ig treated, slope = 0.18, *r*^2^ = 0.017, *p* = 0.343. The data are similar if considered as baseline-normalised C-peptide AUC. (**f**) Baseline *I*.*I*._*359*_ is inversely related to rate (slope) of C-peptide decline over the 24 month study period: placebo treated, slope = −0.18, *r*^2^ = 0.21; *p* = 0.041; CTLA4-Ig treated, slope = −0.003, *r*^2^ = 0.0003, *p* = 0.909. A truncated linear regression was used to estimate the slope of decrease for each individual. Larger negative slope values imply faster decline of stimulated C-peptide. The data are similar if considered as baseline-normalised C-peptide AUC. Black solid circles and solid line, placebo-treated; grey open circles and dotted line, CTLA4-Ig treated

Next, we examined the relationship between the baseline inflammatory/regulatory balance (*I*.*I*._*359*_) and the decline in beta cell function among TN-09 participants. We hypothesised that baseline *I*.*I*._*359*_ might relate to disease activity and correlate with stimulated C-peptide AUC in untreated (placebo) participants. We further hypothesised that the therapeutic effect of CTLA4-Ig would alter the natural disease course, abolishing the relationship established in the placebo arm. A significant inverse relationship was observed between baseline *I*.*I*._*359*_ and the per cent change from baseline C-peptide AUC at 3, 6, 12, 18 and 24 months in placebo-treated participants (*p ≤* 0.015); this relationship was not observed among CTLA4-Ig-treated participants, consistent with immunomodulation altering disease progression (Fig. 2a–e). Similarly, baseline *I*.*I*._*359*_ was inversely related to the rate (slope) of C-peptide decline over the 24 month study in the placebo arm but not in the CTLA4-Ig arm (Fig. 2f).

**Fig. 3:**
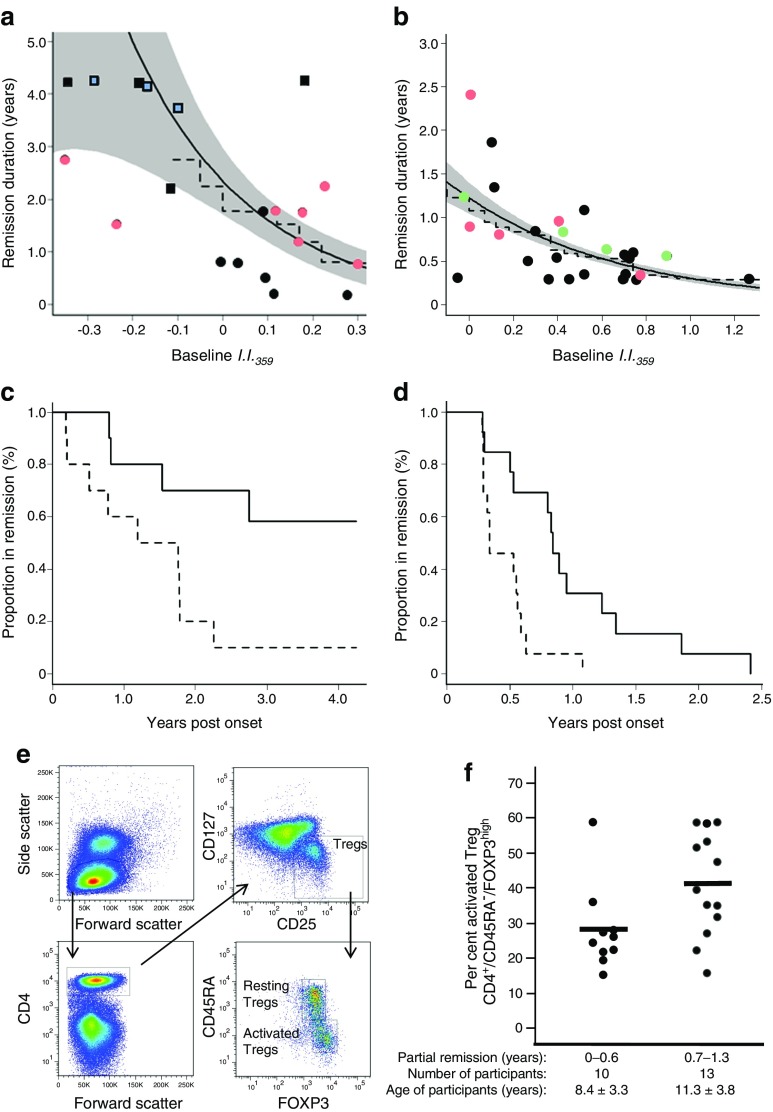
Inflammation levels at onset of type 1 diabetes correlate with duration of the post-onset partial remission. (**a**) Among 19 TN-09 participants in the placebo arm (ages 8–35 years), Weibull (solid line, grey shaded area = 1 SE) and Cox regression (dashed line) analyses identified an inverse relationship between *I*.*I*._*359*_ and median remission duration (*p* = 0.012). The partial remission period was defined as the length of time from diagnosis in which the 2 h stimulated C-peptide AUC was >0.2 nmol/l. (**b**) Weibull (solid line, grey shaded area = 1 SE) and Cox regression (dotted line) analyses identified a significant relationship between *I*.*I*._*359*_ and median remission duration among 26 local individuals with diabetes (*p* = 6.8 × 10^−10^). These participants were aged 4–16 years and possessed high-risk HLA haplotypes; samples were collected 2–7 months after diagnosis (see ESM Table 1 for additional characteristics). As dynamic testing was not performed on the local cohort, the IDAA1c was determined from HbA_1c_ and total daily insulin doses at post-onset clinic visits as described in Mortensen et al [27]. IDAA1c ≤9 is reflective of a stimulated C-peptide >0.3 nmol/l; thus, the remission duration was defined as the last quarterly clinic visit when IDAA1c was ≤9. Among TN-09 participants in the placebo arm, remission lengths determined using the IDAA1c were highly correlated with those determined through dynamic testing (*r* = 0.79); however, as anticipated [49], on average, the use of the IDAA1c underestimated partial remission durations relative to dynamic testing (1.2 ± 1.0 years vs 2.2 ± 1.5 years, respectively). In (**a**, **b**) participant age range is indicated by colour: green, 0–6 years; black, >6–12 years; red, >12–18 years; blue, >18 years; boxes indicate participants in remission at the last visit. (**c**, **d**) Kaplan–Meier analysis: the proportion of TN-09 placebo-arm participants (*n* = 19) (**c**) and local individuals with diabetes (*n* = 26) (**d**) in remission was compared for those with *I*.*I*._*359*_ above the median (dashed line) and those with *I*.*I*._*359*_ below the median (solid line). A logrank test found participants with *I*.*I*._*359*_ above the median were significantly different in both populations (TN-09: *p* = 0.016; local individuals: *p* = 0.005). (**e**) Representative flow cytometry profiles showing the gating strategy for resting and activated Treg populations in cryopreserved PBMCs from local individuals with diabetes. Resting and activated CD4^+^ Tregs were respectively defined as CD45RA^+^/FOXP3^low^ and CD45RA^−^/FOXP3^high^. Since CD45RA and CD45RO are different CD45 isoforms that are respectively expressed on naive and activated/memory T cells, the expression of CD45RO as well as CD25 confirmed the phenotype of resting (CD45RO^−^/CD25^+^) and activated (CD45RO^+^/CD25^high^) Tregs. Each analysis included fluorescence minus one controls to ensure correct gating. (**f**) Per cent activated Tregs among total (active + resting) Tregs in cryopreserved PBMCs collected from 23 local individuals with diabetes during the immediate post-onset period. Eight of these participants were among the 26 analysed by plasma-induced transcription. The data were classified as having shorter or longer partial remissions using the Jenks natural breaks method. The resulting groups had ages of 8.4 ± 3.3 and 11.3 ± 3.8 years, respectively (not significant, *p* > 0.05). The per cent activated Treg was higher in those with longer vs shorter partial remissions (*p* = 0.016). The data are similar if plotted as percentage of activated Treg among total CD4^+^ T cells

To determine whether baseline *I*.*I*._*359*_ is predictive of the duration of the post-onset partial remission period, we investigated the relationship between *I*.*I*._*359*_ and the duration of MMTT-stimulated C-peptide ≥0.2 nmol/l in the TN-09 placebo arm participants (Fig. 3a) and the duration of IDAA1c ≤9 in local individuals with newly diagnosed diabetes (Fig. 3b). In both cohorts, the baseline *I*.*I*._*359*_ was inversely related to the duration of the post-onset partial remission period (*p* ≤ 0.012). Further, participants with *I*.*I*._*359*_ above the median had a significantly shorter duration of persistent insulin secretion than those below the median (Fig. 3c, d), supporting the hypothesis that individuals with higher inflammation at onset will experience accelerated decline in beta cell function. Supporting this observation was the measurement of lower abundances of peripheral activated Tregs (CD4^+^/CD45RA^−^/FOXP3^high^) during the immediate post-onset period in the local participants that exhibited an IDAA1c ≤9 for less than 6 months (Fig. 3e, f; *p* = 0.016).

**Fig. 4:**
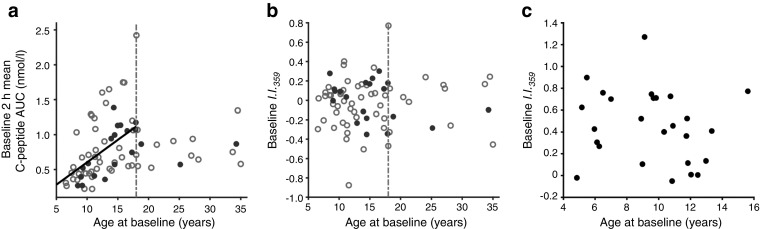
The relationship between *I*.*I*._*359*_ and age of clinical onset. (**a**) Among the 74 TN-09 participants analysed, the age at diagnosis was directly related to baseline 2 h C-peptide AUC (*r*^2^ = 0.28, *p* = 2.1 × 10^−5^). The baseline *I*.*I*._*359*_ was independent of age in TN-09 (**b**) and local (**c**) participants (*n* = 26). In (**a**, **b**), black solid circles, placebo treated (*n* = 19); grey open circles, CTLA4-Ig treated (*n* = 54); dotted/dashed vertical line, 18 years of age

The baseline C-peptide AUC of the TN-09 participants was directly related to age at diagnosis, supporting a recognised relationship [31, 32]. However, baseline *I*.*I*._*359*_ was independent of age in the TN-09 and local cohorts (Fig. 4), indicating that the more rapid decline of beta cell function in those individuals with higher inflammatory bias at baseline was independent of the age of clinical onset. Further, baseline *I*.*I*._*359*_ was independent of time from clinical onset in both cohorts and, in TN-09 participants, there was no correlation between diabetes duration and the baseline stimulated C-peptide AUC.

### Stratification of individuals fosters identification of a CTLA4-Ig therapeutic response signature

Our initial strategy for defining immunomodulation achieved by CTLA4-Ig in TN-09 followed that described in our analyses of the IL-1 antagonism trials [24]. Briefly, induced transcription for each participant at 3, 12 and 24 months was normalised with that of baseline, then differentially induced transcripts between the CTLA4-Ig and placebo arms were identified. A total of 427 differentially induced probe sets (log_2_ ratio >|0.263|, 1.2-fold; ANOVA *p <* 0.05) were identified between the trial arms at ≥1 time point (ESM Fig. 1). Unacceptably, no transcript exhibited an FDR <50% at any time point. We hypothesised that the analysis could be improved by matching treated and placebo participants for baseline *I*.*I*._*359*_ and respectively focusing on those participants with the greatest therapeutic response or the most rapid disease progression.

To identify CTLA4-Ig-treated participants with the greatest therapeutic response, we applied two criteria. The first criterion utilised the placebo arm trend line generated when baseline *I*.*I*._*359*_ was plotted against per cent change from baseline stimulated C-peptide AUC at 3, 6, 12, 18 and 24 months (Fig. 2a–e). Participants who were >1.5 SD above this regression line at ≥3 time points were identified. The second criterion utilised the placebo arm regression line when baseline *I*.*I*._*359*_ was plotted against the rate of C-peptide decline over the 24 month study period (Fig. 2f). Participants who were >1 SD above the trend line were identified. Eight CTLA4-Ig treated participants met both criteria. These eight participants were then compared with placebo participants (*n* = 7) matched for *I*.*I*._*359*_ and residing at or below the placebo regression line depicted in Fig. 2f.

As previously reported, the decrease in beta cell function among CTLA4-Ig-treated participants paralleled that observed in the placebo arm after 6 months [25]. Therefore, we hypothesised that the maximal plasma-induced transcriptional signature representing therapeutic response would be detected at 3 months post-enrolment. In this way, we identified 1509 differentially induced probe sets between the selected eight CTLA4-Ig- and seven placebo-treated participants (mean log_2_ ratio >|0.263|, 1.2-fold; ANOVA *p* < 0.02; FDR ≤30%). These data did not significantly overlap with the cross-sectional data set. Specifically, 50/1374 (3.6%, *χ*^2^ > 0.059) and 10/359 (2.8%, *χ*^2^ = 1) transcripts overlapped those used to define *I*.*I*._*com*_ and *I*.*I*._*359*_, respectively.

**Fig. 5:**
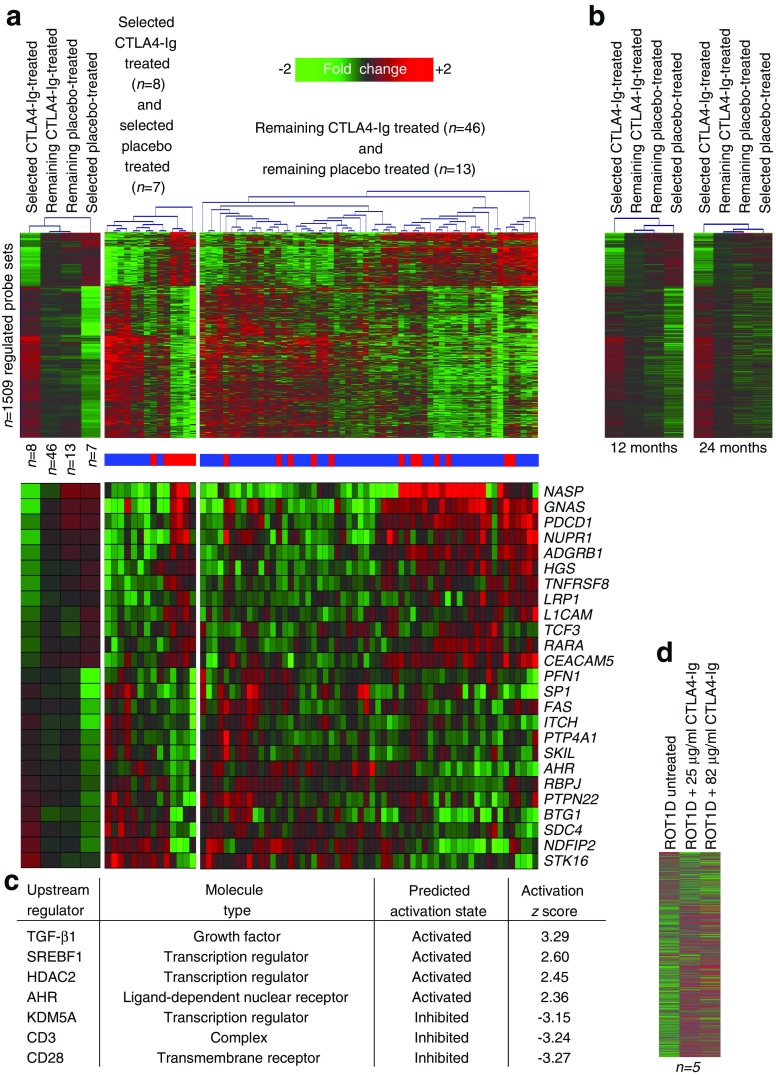
Signature of therapeutic response in TN-09. (**a**) Analysis of the 1509 probe sets regulated between the CTLA4-Ig responders (*n* = 8) and rapidly progressing placebo-treated individuals (*n* = 7) matched for baseline *I*.*I*._*359*_ at the 3 month time point. The top three panels (left to right) illustrate two-way clustering of the selected and excluded participants in each arm. Left panel, mean expression of the four groups. Middle panel, participants selected for the analysis. Right panel, remaining CTLA4-Ig- and placebo-treated participants. Lower three panels, expression levels of a selected set of well-annotated transcripts. The colour bar indicates assigned treatment arm: red, placebo; blue, CTLA4-Ig. Note: samples were not available for two placebo-treated participants at the 3 month time point. The annotated dataset is available from the corresponding author on request. (**b**) Mean expression levels of the 1509 differentially induced probe sets at 12 and 24 months. (**c**) Upstream regulator analysis using IPA. A *z* score >2.0 is significantly activated; a *z* score >−2.0 is significantly inhibited. (**d**) CTLA4-Ig add-back experiment. The mean induced expression levels of the 1509 probe sets after plasma of five local untreated individuals with diabetes were supplemented with 0 μg/ml, 25 μg/ml or 82 μg/ml CTLA4-Ig are shown. ROT1D, recent-onset type 1 diabetes

On average, the signatures of the remaining placebo- and CTLA4-Ig-treated participants were intermediate to those of the selected placebo- and CTLA4-Ig-treated participants and not distinct from one another (Fig. 5a, left). As expected, hierarchical clustering of individual selected placebo- and CTLA4-Ig-treated participants using the 1509 probe sets resulted in distinct grouping (Fig. 5a, middle). In contrast, the remaining participants exhibited imperfect clustering, suggesting that this subgroup possessed both slow-progressing and treatment-non-responder participants (Fig. 5a, right). Consistent with the resumed decline in stimulated C-peptide, at 12 months and 24 months, the plasma milieus of the selected CTLA4-Ig- and placebo-treated participants were more similar, with 236 and 0 probe sets being differentially induced to the aforementioned thresholds, respectively (Fig. 5b).

IPA was then used to identify candidate regulators of the 1509 probe sets differentially induced between the selected CTLA4-Ig- and placebo-treated participants (Fig. 5c). Consistent with immunomodulation anticipated by CTLA4-Ig therapy, IPA identified as being significantly activated (*z* score >2.0): TGF-β1; histone deacetylase co-repressor 2 (HDAC2), a transcriptional repressor that governs NFκB-regulated genes [33]; aryl hydrocarbon receptor (AHR), an important modulator of adaptive responses [34]; and the transcriptional regulator sterol regulatory element-binding protein 1 (SREBF1) [35]. Further, IPA revealed significant inhibition of CD28 and CD3, molecules important in T cell activation, and lysine demethylase 5A (KDM5A), important in natural killer cell activation [36], (*z* score <−2.0) in CTLA4-Ig treated individuals. Transcripts annotated under these candidate upstream regulators are known to possess roles in attenuating adaptive immune responses (*SKIL*, *SMAD2*, *PTPN22*, *AHR*), mediating cell adhesion (*CEACAM5*, *L1CAM*) and regulating proliferation/apoptosis (*SDC4*, *BAX*, *NDFIP2*, *BTG1*).

The half-life of CTLA4-Ig is 14 days. At the 3 month sampling, the TN-09 dosing schedule was such that participants had last received an infusion 4 weeks prior; as such, it was considered unlikely there would be residual CTLA4-Ig in the samples. We reasoned that if the treatment arm signature was a direct consequence of carry-over CTLA4-Ig in the plasma, it would be possible to recapitulate that signature in samples of untreated individuals by spiking the cultures with CTLA4-Ig. Plasma of untreated individuals with diabetes was therefore supplemented with 0 μg/ml, 25 μg/ml (estimated steady-state trough level) or 82 μg/ml CTLA4-Ig (estimated steady-state high level). Even at the highest level, only 880 (58.3%) of the 1509 probe sets differentially induced between the selected CTLA4-Ig- and placebo-treated individuals were directionally concordant, and only 67 (4.4%) were directionally concordant and possessed an FDR <30% (Fig. 5d). This indicates that signatures of the participants in the treatment arm were largely independent of carry-over CTLA4-Ig and that signatures of the selected CTLA4-Ig-treated participants were reflective of treatment-mediated immunomodulation.

### Variation in baseline signatures defines distinct subgroups that differentially respond to CTLA4-Ig

**Fig. 6:**
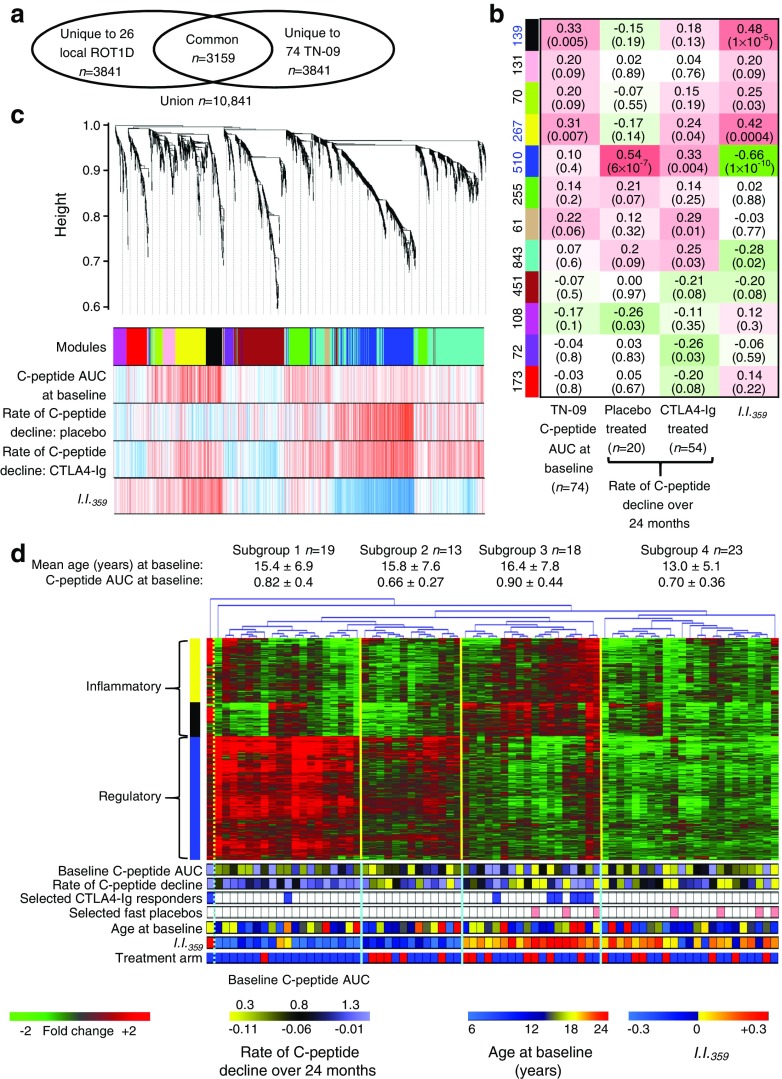
Analysis of highly variant transcripts in baseline signatures indicates the existence of type 1 diabetes subgroups. (**a**) Identification of highly variant transcripts common to TN-09 participants (*n* = 74) and local individuals with diabetes (*n* = 26). The Affymetrix U133 plus 2.0 array has probe sets for interrogation of >47,000 transcripts. The 20,000 probe sets that, on average, exhibited the lowest signal intensity were filtered from the analysis (maximum log_2_ intensity <4 RFU). The 7000 probe sets exhibiting the greatest median absolute deviation were retained. (**b**) Co-expression networks among the 3159 commonly variant transcripts were identified with WGCNA using a power of *β* = 12 (linear regression model fitting index *R*^2^ > 0.8). Twelve co-expression modules were identified (represented by rows, labelled by colour). The number of probe sets assigned to each module is indicated; 79/3159 (2.5%) could not be assigned to a co-expression network. Columns represent a trait or cohort subset. The correlation coefficient shown within each box is between the module eigengene and the trait or cohort; the correlation *p* value is given in parentheses. An eigengene is defined as the first principal component of a given module and is representative of the expression profiles of genes within that module. Red represents a positive correlation between the module eigengene and trait; green represents a negative correlation. Significant relationships were not detected with age, sex, Tanner stage, HbA_1c_ at baseline, HLA, autoantibody status, BMI, duration of disease or complete blood cell counts. (**c**) Dendrogram illustrating co-expression modules. Each vertical line corresponds to a gene. The *y*-axis indicates the network distance (1 – topological overlap), values closer to 0 indicate greater similarity of transcript expression profiles across samples. Co-expression modules are indicated by colours in the first colour band. The additional colour bands illustrate the positively correlated (progressively red) or negatively correlated (progressively blue) transcripts for the trait/cohorts. (**d**) Hierarchical clustering of the baseline signatures of TN-09 participants using the 916 significantly regulated transcripts belonging to the three modules significantly correlated with 2 h AUC at baseline and the rate of C-peptide decline within the placebo-treated arm. The three modules included 85 transcripts used to calculate *I*.*I*._*359*_ (85/359, 23.7%, *χ*^2^ < 1.0 × 10^−4^). Four major subgroups of individuals with new onset diabetes are indicated, none of which significantly differ by age or baseline C-peptide AUC. Colour bars indicate: fold change, baseline C-peptide AUC, rate of C-peptide decline over 24 months, selected CTLA4-Ig responders (as in Fig. 5; blue), selected rapidly progressing placebos (as in Fig. 5; ‘Selected fast placebos’; peach), age at baseline and *I.I.*_*359*_. The colour bar indicates assigned treatment arm: red, placebo; blue, CTLA4-Ig. The 3159 commonly variant probe sets and the modules to which they were assigned are available as an annotated dataset from the corresponding author on request

The baseline *I*.*I*._*359*_ was significantly related to post-onset C-peptide AUC and was useful for matching participants in analyses aimed at defining the therapeutic effects of CTLA4-Ig. However, *I*.*I*._*359*_, which is derived from cross-sectional analyses, did not completely differentiate CTLA4-Ig treatment responders from non-responders, suggesting that *I*.*I*._*359*_ does not entirely capture the variation among newly diagnosed individuals. Therefore, we identified the 3159 most variant transcripts common to the local and TN-09 participants at baseline (Fig. 6a). This dataset was analysed with WGCNA, a software that clusters the main patterns of variation into modules of co-expressed transcripts and correlates these to phenotypes.

Among the 3159 transcripts, WGCNA identified 12 modules. Two modules significantly correlated with the baseline C-peptide AUC among TN-09 participants (black and yellow); a third module (blue) correlated with the rate of C-peptide decline among both placebo and CTLA4-Ig treated participants (Fig. 6b, c). These modules also correlated with *I*.*I*._*359*_, suggesting that the inflammatory/regulatory dynamic previously identified by cross-sectional analysis is also present among newly diagnosed individuals.

**Fig. 7:**
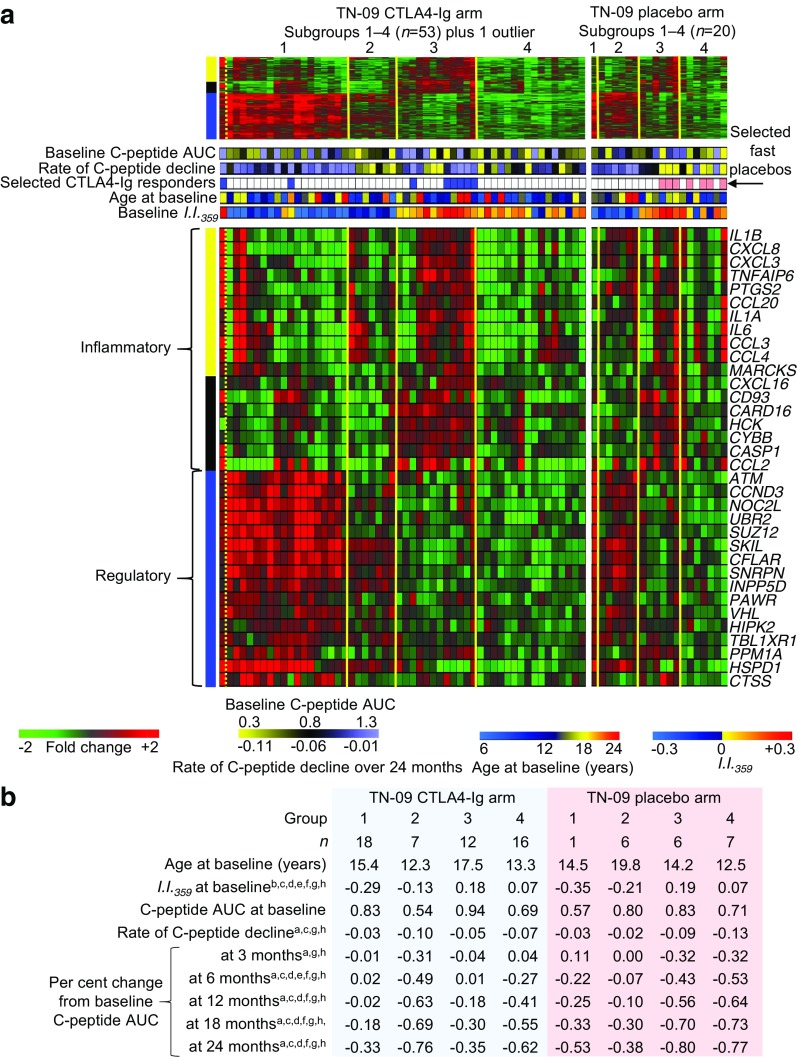
Type 1 diabetes subgroups exhibit different responses to CTLA4-Ig treatment. (**a**) Heatmaps illustrating expression levels of the 916 significantly regulated transcripts belonging to the three modules significantly correlated with 2 h C-peptide AUC at baseline and the placebo-treated arm of TN-09; an annotated heatmap is shown below. The four major subgroups (as shown in Fig. 6d) are indicated for each arm. Well-annotated transcripts are illustrated, and the module to which they belong is indicated by the colour bar on the left. Colour bars are provided that indicate: fold change; baseline C-peptide AUC; rate of C-peptide decline over 24 months; selected CTLA4-Ig responders (as in Fig. 5; blue); selected placebos (as in Fig. 5; ‘Selected fast placebos’; peach); age at baseline; and *I*.*I*._*359*_. (**b**) TN-09 participants belonging to subgroups 1–4 exhibit different responses to CTLA4-Ig treatment. Tabulated are subgroup mean values for each metric. Statistical differences were assessed with a two-tailed unpaired Student’s *t* test, superscript letters denote *p* < 0.05 for: ^a^CTLA4-Ig subgroup 1 vs CTLA4-Ig subgroup 2; ^b^CTLA4-Ig subgroup 1 vs CTLA4-Ig subgroup 3; ^c^CTLA4-Ig subgroup 1 vs CTLA4-Ig subgroup 4; ^d^CTLA4-Ig subgroup 2 vs CTLA4-Ig subgroup 3; ^e^CTLA4-Ig subgroup 2 vs CTLA4-Ig subgroup 4; ^f^CTLA4-Ig subgroup 3 vs placebo subgroup 3; ^g^placebo subgroup 2 vs placebo subgroup 3; and ^h^placebo subgroup 2 vs placebo subgroup 4

To investigate whether the immune activity represented by these three modules could define subgroups among newly diagnosed individuals, the baseline signatures of TN-09 participants were subjected to hierarchical clustering. This identified four major subgroups that did not significantly differ by age or baseline C-peptide AUC (Fig. 6d). For the 916 transcripts encompassed by these modules, IPA identified numerous candidate upstream regulators, including lipopolysaccharide (1.2 × 10^−31^), IL-1B (1.7 × 10^−18^), TNF (2.4 × 10^−29^), IFN-γ (7.7 × 10^−19^), IL-10 (7.7 × 10^−13^) and TGF-β1 (1.8 × 10^−8^). The yellow and black modules included numerous proinflammatory annotations, including cytokines (*IL1A*, *IL1B*, *IL6*) and chemokines (*CCL2*, *CCL3*, *CCL4*); conversely; the blue module included many transcripts related to IL-10 and TGF-β signalling (*SKI*, *SKIL*, *INPP5D*, *SUZ12*) (Fig. 7a).

The CTLA4-Ig and placebo arms of TN-09 were independently analysed from the perspective of these four subgroups. While the limited number of participants assigned to these subgroups precludes robust conclusions, significant age-independent differences were identified in the rate of C-peptide decline within each treatment arm. Among CTLA4-Ig-treated participants, those in subgroup 1 exhibited the smallest per cent change from baseline C-peptide AUC at 3, 12, 18 and 24 months and the lowest overall rate of C-peptide decline over 24 months compared with those in the other three subgroups (Fig. 7b). The CTLA4-Ig-treated participants assigned to subgroup 2 were significantly younger than their placebo-treated counterparts (Kolmogorov–Smirnov test, *p* < 0.04), and exhibited a faster rate of C-peptide decline and a greater per cent change from baseline C-peptide AUC at 3, 6, 12, 18 and 24 months than the other subgroups. Among the placebo-treated participants, the rate of C-peptide decline and per cent change from baseline C-peptide AUC for subgroups 3 and 4 were significantly greater than those of subgroup 2 (and greater than the single placebo-treated individual assigned to subgroup 1). Finally, within subgroup 3, CTLA4-Ig treatment significantly reduced the per cent change from baseline C-peptide AUC at 6, 18 and 24 months when compared with the placebo arm (Fig. 7b).

**Fig. 8:**
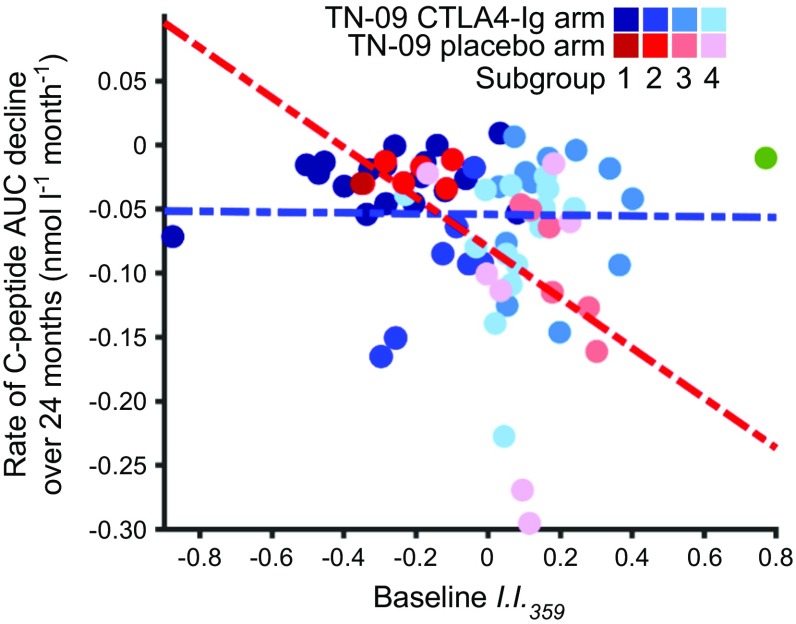
Type 1 diabetes subgroups exhibit different rates of C-peptide decline and responses to CTLA4-Ig treatment. A significant inverse relationship between baseline *I*.*I*._*359*_ and the rate (slope) of C-peptide decline in placebo-treated TN-09 participants is described in Fig. 2f. Here, this relationship is considered from the perspective of subgroups 1–4. The green data point is the outlying CTLA4-Ig participant not assigned to a subgroup (left-most participant in Fig. 6d). Regression lines are shown for the CTLA4-Ig and placebo arms of TN-09 in blue and red, respectively

These data suggest that baseline plasma-induced signatures can define type 1 diabetes subgroups. Participants in subgroups 1 and 2 exhibited greater baseline regulatory bias (and lower *I*.*I*._*359*_) and a generally slower rate of C-peptide decline. Conversely, participants in subgroups 3 and 4 exhibited greater innate inflammatory bias and a faster rate of C-peptide decline. Subgroup 3 contained most of the selected CTLA4-Ig responders and rapidly progressing individuals who received placebo. The data also suggest that participants with greater baseline inflammatory activity (subgroups 3 and 4) exhibited a better therapeutic response to CTLA4-Ig during the 24 month study period (Fig. 8).

## Discussion

We find that individuals with type 1 diabetes possessing lower innate inflammatory bias at the time of clinical onset, as measured through plasma-induced transcription, are more likely to have slower rates of decline in residual insulin secretion. This conclusion was supported through two independent analyses: one based on a scoring system developed from cross-sectional analyses of individuals recently diagnosed with diabetes and healthy control individuals (*I*.*I*._*359*_); and a second that utilised WGCNA to identify clinically relevant highly variant transcripts among newly diagnosed volunteers. We also associated longer persistence of IDAA1c ≤9 with higher peripheral abundances of activated Treg, corroborating Moya et al [37]. These analyses contribute to a growing effort to predict disease progression in type 1 diabetes [26, 37–41] and are in line with studies linking elevated innate inflammation with diabetes progression [42–45]. Importantly, they support the existence of subgroups that differ in inflammatory/regulatory balance, rate of post-onset disease progression and responsiveness to therapeutic intervention with CTLA4-Ig.

These findings add to an understanding of phenotypic heterogeneity among newly diagnosed individuals. Arif et al described two distinct adaptive immune response patterns among newly diagnosed children and adolescents: (1) a partially regulated IL-10-dominated CD4 T lymphocyte response; and (2) a proinflammatory IFN-γ-dominated CD4 T lymphocyte response [46]. Similar to our analysis, and possibly paralleling subgroups 1–2 and 3–4, individuals exhibiting regulatory vs inflammatory bias were equally distributed.

Two distinct insulitic profiles have been observed in people with new-onset type 1 diabetes, further supporting the existence of subtypes [46, 47]. Individuals diagnosed below 7 years of age possessed islets with lower insulin content and greater numbers of infiltrating immunocytes, in particular CD20^+^ B cells. Individuals diagnosed after 13 years of age possessed islets with greater insulin content, fewer infiltrating immunocytes and low abundances of CD20^+^ B cells. Participants of intermediate age exhibited either one profile or the other [47]. In contrast, the subgroups defined herein did not exhibit age dependence. However, among the studied TN-09 participants, only two were diagnosed under 7 years of age; these were ascribed to subgroups 3 and 4, which exhibited inflammatory bias and a more rapid rate of C-peptide decline.

Our study of the AIDA trial [24] suggested that individuals with diabetes with the highest levels of baseline inflammation may have benefited the most from IL-1Ra treatment, as they tended to experience the greatest reduction in inflammation and retained the most beta cell function. Likewise, our findings suggest that participants with greater inflammatory bias (higher baseline *I*.*I*._*359*_, and ascribed primarily to subgroup 3, both of which are associated with more rapid disease progression) benefited more from CTLA4-Ig treatment. One possible explanation for this observation is that participants possessing greater regulatory bias have disease activity too modest to be therapeutically altered during the trial. Whatever the biological basis, heterogeneity in disease progression among individuals is an obstacle in clinical trials, as inclusion of slow progressors impairs the ability to detect treatment-specific changes in C-peptide production.

Stratification of participants by baseline *I*.*I*._*359*_ facilitated our analysis of the therapeutic response to CTLA4-Ig. The original analysis of TN-09 showed that CTLA4-Ig treatment delayed the decline of beta cell function [25]. Here, selected CTLA4-Ig responders exhibited signatures indicative of CD28 and CD3 inhibition, an observation consistent with co-stimulation blockade and impaired activation of auto-aggressive T cells and in line with the presumed mechanism of action of CTLA4-Ig. As demonstrated by TN-09, CTLA4-Ig treatment can suppress inflammation after disease is established, suggesting the possibility of alternative mechanisms beyond the prevention of naive T cell activation through co-stimulation blockade. In mice, CTLA4-Ig mediates regulation of activated effector T cells through a nitric oxide/Treg/TGF-β-dependent pathway [48]. Our analysis identified TGF-β1 as a potential regulator of the immunoregulatory-biased signature of the selected CTLA4-Ig- treated participants, suggesting that such alternative mechanisms may be associated with human responses.

Our findings have important implications for the stratification of individuals with diabetes. The ability to identify individuals with rapidly progressing disease would allow for more informative and targeted trials of participants most likely to benefit from therapeutic intervention. The size of this study is a limitation. Additional studies are needed to determine if the subgroups defined here are seen in other cohorts, whether they are governed by genetic and/or environmental factors and whether they differentiate progression rates prior to diabetes onset, particularly in multiple-antibody-positive ‘at-risk’ participants at earlier (preclinical) states of the disease.

## Electronic supplementary material

ESM (PDF 434 kb)

## Data Availability

Gene expression data files are publicly available through the National Center for Biotechnology Information Gene Expression Omnibus (accession number GSE102234).

## Abbreviations

AHR: Aryl hydrocarbon receptor
AIDA: Anti-Interleukin-1 in Diabetes Action
CTLA4-Ig: Cytotoxic T lymphocyte-associated protein 4/Fc fraction of the IgG_1_ fusion protein (abatacept)
FDR: False-discovery rate
HDAC2: Histone deacetylase co-repressor 2
IDAA1c: Insulin-dose-adjusted A_1c_
*I*.*I*._*com*_: Composite inflammatory index (based on 1374 transcripts)
*I*.*I*._*359*_: Inflammatory index based on 359 transcripts
IL-1Ra: IL-1 receptor antagonist
IPA: Ingenuity Pathway Analysis
KDM5A: Lysine demethylase 5A
MMTT: Mixed meal tolerance test
PBMC: Peripheral blood mononuclear cell
ROC: Receiver operator characteristic
SREBF1: Sterol regulatory element-binding protein 1
TN-09: TrialNet CTLA4-Ig (Abatacept) trial
Treg: Regulatory T cell
WGCNA: Weighted gene co-expression network analysis
