# Supplementary material for: Innate immune activity as a predictor of persistent insulin secretion and association with responsiveness to CTLA4-Ig treatment in recent-onset type 1 diabetes
Source: Diabetologia. 2018 Aug 30;61(11):2356–70. doi: 10.1007/s00125-018-4708-x (PMC6182660; doi:10.1007/s00125-018-4708-x)
Supplement: Supplementary file 1 — (PDF 434 kb) [file 125_2018_4708_MOESM1_ESM.pdf]

ESM TABLE 1 Subject Information TN09

Study	Treatment Arm	Subject ID	Gender	Age at diagnosis	Age at baseline visit	Duration of DM @ visit	I.I.359	Length of Partial remission years (CPEP > 0.2 nmol/L)	HbA1C at initial visit (mmol/mol)	HbA1C at initial visit (%)	Weight (kg)	Height (cm)	BMI SDS	BMI Percen-tile	Number of Detected Autoanti-bodies	HLA Risk (High: DR3 and/ or DR4 positive; Low nonDR3 and nonDR4)	WGCNA Group Assign-ment	Selected Treated Responder Sample and Placebo Fast Sample	Mean 2hr CPEP by min (nmol/L) Baseline	Mean 2hr CPEP by min (nmol/L) Day 84	Mean 2hr CPEP by min (nmol/L) Day 196	Mean 2hr CPEP by min (nmol/L) Day 364	Mean 2hr CPEP by min (nmol/L) Day 560	Mean 2hr CPEP by min (nmol/L) Day 728
TN09	ABATACEPT	CTLA4_CT_Pt9002922	Male	17.91	18.21	0.30	-0.33	NA	40.3	6.5	78.6	170	1.32	91	1	high	G1	0	1.07	1.00	0.93	1.11	1.00	1.04
TN09	ABATACEPT	CTLA4_CT_Pt9003388	Female	21.08	21.35	0.27	-0.02	NA	32.9	5.3	81.2	182.5	0.31	62	2	high	G2	0	0.86	0.54	0.42	0.36	0.30	0.12
TN09	ABATACEPT	CTLA4_CT_Pt9009315	Male	11.59	11.85	0.27	-0.09	NA	39.1	6.3	38	147	-0.05	48	3	high	G2	0	0.68	0.46	0.36	0.24	0.19	0.15
TN09	ABATACEPT	CTLA4_CT_Pt9010058	Male	14.21	14.48	0.27	0.16	NA	32.9	5.3	68.2	180.8	0.48	68	4	high	G4	0	0.60	0.47	0.43	0.33	0.24	0.19
TN09	ABATACEPT	CTLA4_CT_Pt9011880	Female	9.28	9.57	0.29	-0.13	NA	45.3	7.3	36.5	134.7	1.17	88	2	high	G2	0	0.22	0.19	0.12	0.10	0.11	0.03
TN09	ABATACEPT	CTLA4_CT_Pt9015071	Male	16.52	16.73	0.21	-0.05	NA	39.1	6.3	67.2	174.6	0.38	65	2	high	G2	0	0.55	0.27	0.16	0.06	0.07	0.05
TN09	ABATACEPT	CTLA4_CT_Pt9016746	Female	8.11	8.29	0.18	0.14	NA	35.3	5.7	35.6	139	1.01	84	3	high	G4	0	1.03	0.90	0.90	0.59	0.72	0.72
TN09	ABATACEPT	CTLA4_CT_Pt9018458	Male	8.38	8.66	0.28	0.05	NA	39.1	6.3	27.9	138.5	-1.03	15	3	low	G4	0	0.44	0.53	0.41	0.22	0.25	0.21
TN09	ABATACEPT	CTLA4_CT_Pt9019691	Female	6.51	6.67	0.17	0.02	NA	39.7	6.4	21.8	116.1	0.48	68	2	high	G4	0	0.27	0.36	0.10	0.10	0.03	0.01
TN09	ABATACEPT	CTLA4_CT_Pt9019970	Female	10.02	10.24	0.22	0.06	NA	50.2	8.1	49.5	145	1.7	96	3	high	G4	0	0.71	0.66	0.41	0.25	0.20	0.15
TN09	ABATACEPT	CTLA4_CT_Pt9027000	Male	14.98	15.24	0.26	0.06	NA	36.0	5.8	67.5	178.5	0.4	66	4	high	G4	0	0.81	0.85	0.85	0.63	0.48	0.50
TN09	ABATACEPT	CTLA4_CT_Pt9031301	Female	11.13	11.28	0.15	-0.26	NA	40.9	6.6	37.9	157.4	-1.13	13	3	high	G2	0	0.68	0.50	0.29	0.21	0.03	0.03
TN09	ABATACEPT	CTLA4_CT_Pt9033311	Female	8.15	8.42	0.27	0.05	NA	44.6	7.2	29.7	131.6	0.52	70	2	high	G3	0	0.35	0.24	0.21	0.09	0.05	0.02
TN09	ABATACEPT	CTLA4_CT_Pt9034909	Male	10.35	10.63	0.27	-0.23	NA	42.8	6.9	59.7	157.7	1.83	97	3	low	G4	0	1.29	1.39	1.10	1.23	0.98	0.87
TN09	ABATACEPT	CTLA4_CT_Pt9034944	Male	34.78	35.00	0.22	-0.45	NA	41.5	6.7	83	176.6	0.1	54	3	high	G1	0	0.58	0.75	0.66	0.75	0.66	0.57
TN09	ABATACEPT	CTLA4_CT_Pt9035621	Male	17.25	17.50	0.25	0.05	NA	36.6	5.9	86.8	175	1.61	95	2	high	G3	0	1.06	0.71	0.88	0.61	0.62	0.52
TN09	ABATACEPT	CTLA4_CT_Pt9039251	Male	13.45	13.66	0.21	0.17	NA	37.8	6.1	46.4	164.5	-0.8	21	3	low	G4	0	0.51	0.47	0.38	0.37	0.28	0.28
TN09	ABATACEPT	CTLA4_CT_Pt9040109	Female	15.85	16.06	0.21	-0.14	NA	39.7	6.4	112.1	169.9	2.33	99	2	high	G1	0	1.75	1.12	1.40	1.61	1.30	1.49
TN09	ABATACEPT	CTLA4_CT_Pt9041256	Female	20.18	20.44	0.26	0.03	NA	36.6	5.9	61.7	161.3	0.08	53	4	low	G1	CT_8Resp	0.53	0.39	NA	NA	NA	0.57
TN09	ABATACEPT	CTLA4_CT_Pt9042186	Female	13.76	13.98	0.22	-0.50	NA	47.1	7.6	42.5	159	-1.06	15	3	high	G1	0	0.49	0.57	0.61	0.56	0.50	0.38
TN09	ABATACEPT	CTLA4_CT_Pt9043824	Male	17.75	18.02	0.27	0.77	NA	34.7	5.6	72.5	169	0.98	84	3	high	OL	CT_8Resp	2.42	2.03	2.19	1.90	1.87	2.53
TN09	ABATACEPT	CTLA4_CT_Pt9045146	Female	7.50	7.77	0.27	0.08	NA	44.6	7.2	34.2	129.7	1.63	95	3	high	G1	0	0.53	0.52	0.54	0.38	0.23	0.28
TN09	ABATACEPT	CTLA4_CT_Pt9048858	Male	8.93	9.18	0.25	-0.28	NA	34.1	5.5	30.8	129.2	0.91	82	4	high	G1	0	0.67	0.49	0.34	0.34	NA	0.20
TN09	ABATACEPT	CTLA4_CT_Pt9051989	Male	7.48	7.73	0.25	0.04	NA	40.9	6.6	28.8	122	1.54	94	4	low	G4	0	0.36	0.17	0.11	0.03	0.01	0.01
TN09	ABATACEPT	CTLA4_CT_Pt9052289	Female	23.78	24.04	0.26	0.24	NA	30.4	4.9	60.5	160.1	0.2	57	1	high	G4	0	0.91	0.92	0.71	0.55	0.40	0.22
TN09	ABATACEPT	CTLA4_CT_Pt9053269	Male	11.10	11.36	0.25	-0.34	NA	33.5	5.4	44.9	142.9	1.37	92	2	high	G1	0	1.08	0.96	0.76	0.52	0.27	0.24
TN09	ABATACEPT	CTLA4_CT_Pt9055573	Male	17.08	17.35	0.27	-0.19	NA	40.9	6.6	93	181	1.63	95	3	high	G1	0	1.20	1.01	1.17	1.09	0.82	0.77
TN09	ABATACEPT	CTLA4_CT_Pt9059514	Female	17.74	17.99	0.25	-0.47	NA	37.8	6.1	55.2	166.3	-0.46	32	4	low	G1	0	0.71	0.70	0.63	NA	NA	0.25
TN09	ABATACEPT	CTLA4_CT_Pt9060795	Male	17.02	17.29	0.27	-0.28	NA	34.7	5.6	79.5	174	1.27	90	2	high	G1	0	0.56	0.74	0.96	0.90	0.68	0.49
TN09	ABATACEPT	CTLA4_CT_Pt9060824	Female	11.18	11.40	0.23	0.20	NA	NA	NA	42.1	154.9	-0.06	48	3	low	G3	0	0.78	0.86	0.40	0.26	0.14	0.04
TN09	ABATACEPT	CTLA4_CT_Pt9064499	Male	33.43	33.70	0.27	0.17	NA	32.2	5.2	70.5	172.1	-0.44	33	2	low	G3	CT_8Resp	0.75	1.01	1.23	0.97	0.74	0.71
TN09	ABATACEPT	CTLA4_CT_Pt9066427	Female	7.30	7.47	0.17	-0.21	NA	42.8	6.9	24.2	123.3	0.18	57	2	high	G1	0	0.45	0.49	0.65	0.50	0.42	0.37
TN09	ABATACEPT	CTLA4_CT_Pt9068024	Male	10.54	10.81	0.27	0.40	NA	45.9	7.4	46.5	141.5	1.68	95	1	high	G3	CT_8Resp	0.22	0.24	0.22	NA	0.11	0.06
TN09	ABATACEPT	CTLA4_CT_Pt9068424	Male	34.29	34.50	0.21	0.24	NA	34.1	5.5	84.4	191.4	-0.64	26	2	low	G3	CT_8Resp	1.35	1.55	1.74	1.56	1.36	1.43
TN09	ABATACEPT	CTLA4_CT_Pt9072101	Male	9.61	9.77	0.16	-0.06	NA	42.2	6.8	29.1	141.5	-1.3	10	1	high	G1	0	0.44	0.70	0.76	0.64	0.49	0.46
TN09	ABATACEPT	CTLA4_CT_Pt9074578	Female	12.21	12.46	0.25	-0.12	NA	32.2	5.2	44.3	147	0.66	75	3	high	G1	0	1.60	1.58	1.33	1.28	NA	0.99
TN09	ABATACEPT	CTLA4_CT_Pt9082597	Male	26.93	27.19	0.26	0.12	NA	28.5	4.6	87	190.9	-0.18	43	3	high	G3	0	0.94	0.57	0.67	0.81	0.33	0.52
TN09	ABATACEPT	CTLA4_CT_Pt9083384	Male	10.42	10.69	0.27	0.10	NA	40.3	6.5	40	141.2	1.08	86	3	high	G3	CT_8Resp	0.47	0.60	0.75	0.51	0.48	0.43
TN09	ABATACEPT	CTLA4_CT_Pt9084843	Male	15.40	15.67	0.27	0.34	NA	36.6	5.9	74.3	175	1.11	87	4	high	G3	CT_8Resp	0.67	0.87	0.66	0.32	0.59	0.56
TN09	ABATACEPT	CTLA4_CT_Pt9084986	Female	10.55	10.76	0.21	0.36	NA	36.6	5.9	61.5	155.3	1.87	97	4	high	G3	0	1.27	1.48	1.79	1.74	1.52	1.03
TN09	ABATACEPT	CTLA4_CT_Pt9086296	Male	12.88	13.07	0.19	0.15	NA	36.6	5.9	51.5	158	0.072	76	4	high	G4	0	1.45	1.28	1.19	0.92	0.98	0.80
TN09	ABATACEPT	CTLA4_CT_Pt9087810	Male	8.77	9.03	0.26	-0.04	NA	36.0	5.8	28	131.1	0.07	53	2	high	G2	0	0.50	0.53	0.46	0.53	0.40	0.49
TN09	ABATACEPT	CTLA4_CT_Pt9088623	Male	11.32	11.58	0.27	-0.88	NA	45.3	7.3	31.1	136.6	-0.41	34	2	high	G1	0	0.51	0.42	0.28	0.16	0.10	0.06
TN09	ABATACEPT	CTLA4_CT_Pt9088950	Female	12.55	12.82	0.27	-0.17	NA	38.4	6.2	56.8	157.4	1.14	87	2	high	G1	0	0.90	0.81	1.03	1.14	0.78	0.47
TN09	ABATACEPT	CTLA4_CT_Pt9088997	Male	NA	15.85	NA	0.07	NA	36.0	5.8	81.72	170.7	1.73	96	3	low	G3	CT_8Resp	1.75	1.65	1.45	1.75	1.69	2.63
TN09	ABATACEPT	CTLA4_CT_Pt9090498	Female	6.37	6.54	0.17	-0.30	NA	44.0	7.1	25.6	126.1	0.46	68	4	high	G2	0	0.31	0.11	0.12	0.02	0.02	0.01
TN09	ABATACEPT	CTLA4_CT_Pt9091454	Male	10.95	11.19	0.25	-0.40	NA	36.0	5.8	44.1	148	0.99	84	2	high	G1	0	1.24	0.86	0.73	0.62	0.58	0.44
TN09	ABATACEPT	CTLA4_CT_Pt9092010	Male	13.36	13.59	0.23	0.03	NA	34.7	5.6	54.6	162.3	0.63	74	1	low	G3	0	1.65	0.32	1.28	0.95	1.11	0.64
TN09	ABATACEPT	CTLA4_CT_Pt9092037	Female	11.84	12.07	0.23	-0.03	NA	32.2	5.2	38.8	143	0.3	62	4	high	G4	0	0.60	0.38	0.13	0.39	0.19	0.13
TN09	ABATACEPT	CTLA4_CT_Pt9092765	Male	18.04	18.28	0.24	0.12	NA	42.8	6.9	73.54	1.76	0.52	70	2	high	G4	0	0.55	0.48	0.36	0.34	0.25	0.21
TN09	ABATACEPT	CTLA4_CT_Pt9093838	Female	26.68	26.95	0.27	0.16	NA	NA	NA	52.8	159.4	-0.77	22	2	high	G4	0	0.70	0.48	0.59	0.40	0.43	0.43
TN09	ABATACEPT	CTLA4_CT_Pt9095979	Female	10.53	10.81	0.27	-0.01	NA	40.3	6.5	31.3	144.9	-1.24	11	3	high	G4	0	0.40	1.22	0.39	0.27	0.14	0.14
TN09	ABATACEPT	CTLA4_CT_Pt9097442	Female	10.10	10.35	0.26	0.08	NA	42.8	6.9	33.2	135.8	0.37	64	2	high	G4	0	0.44	0.36	0.38	0.33	0.21	0.06
TN09	ABATACEPT	CTLA4_CT_Pt9098085	Male	27.86	28.12	0.26	-0.26	NA	38.4	6.2	80.9	182.7	-0.15	44	2	high	G1	0	0.64	0.74	0.73	1.02	0.93	0.69
TN09	PLACEBO	CTLA4_PL_Pt9001411	Male	8.98	9.23	0.25	0.11	0.2	39.1	6.3	32.90	132.50	1.03	85	4	high	G4	PL_7Fast	0.27	0.13	0.07	0.01	0.01	0.01
TN09	PLACEBO	CTLA4_PL_Pt9003177	Female	14.27	14.48	0.22	-0.18	4.21	34.7	5.6	56.20	171.20	-0.15	44	3	high	G2	0	1.00	0.87	0.73	0.76	0.67	0.72
TN09	PLACEBO	CTLA4_PL_Pt9003276	Male	17.66	17.88	0.22	-0.35	4.23	35.3	5.7	83.40	179.93	1.08	86	2	high	G2	0	1.07	1.18	1.02	0.96	0.65	0.73
TN09	PLACEBO	CTLA4_PL_Pt9003652	Female	12.78	12.91	0.14	-0.23	1.53	56.4	9.1	39.40	150.00	-0.45	33	1	low	G2	0	0.36	0.39	0.44	0.37	0.29	0.27
TN09	PLACEBO	CTLA4_PL_Pt9009476	Male	10.91	11.17	0.27	0.03	0.79	36.6	5.9	47.90	155.70	0.89	81	3	high	G4	PL_7Fast	0.41	0.29	0.22	0.09	0.04	0.01
TN09	PLACEBO	CTLA4_PL_Pt9012575	Male	9.05	9.33	0.27	0.09	0.51	44.6	7.2	32.00	134.60	0.63	74	3	high	G4	PL_7Fast	0.52	0.21	0.09	0.01	0.01	0.01
TN09	PLACEBO	CTLA4_PL_Pt9020619	Female	14.78	14.99	0.21	0.17	1.19	48.4	7.8	79.80	165.80	1.75	96	2	high	G3	0	1.13	0.71	0.52	0.81	0.17	0.37
TN09	PLACEBO	CTLA4_PL_Pt9022509	Male	13.72	13.93	0.21	-0.12	2.21	39.7	6.4	56.93	161.00	0.92	82	4	high	G2	0	0.94	0.76	0.92	0.66	0.43	0.44
TN09	PLACEBO	CTLA4_PL_Pt9031867	Male	18.64	18.81	0.17	-0.17	4.13	38.4	6.2	90.50	181.00	1.32	91	2	high	G4	0	0.87	0.91	NA	0.49	0.57	0.52
TN09	PLACEBO	CTLA4_PL_Pt9034582	Male	8.82	9.02	0.19	0.00	0.81	47.7	7.7	26.40	130.70	-0.43	34	4	high	G4	PL_7Fast	0.40	0.30	0.28	0.15	0.10	0.06
TN09	PLACEBO	CTLA4_PL_Pt9034808	Male	24.92	25.16	0.24	-0.29	4.25	47.7	7.7	57.00	177.00	-1.64	5	3	low	G2	0	0.57	0.44	0.32	0.31	0.25	0.33
TN09	PLACEBO	CTLA4_PL_Pt9037347	Male	NA	17.30	NA	0.12	1.99	40.9	6.6	73.20	180.00	0.39	65	3	NA	G3	0	0.75	NA	0.61	0.40	0.44	0.27
TN09	PLACEBO	CTLA4_PL_Pt9047617	Male	14.21	14.48	0.27	-0.35	2.75	37.8	6.1	47.90	174.70	-2.03	2	3	high	G1	0	0.57	0.64	0.45	0.43	0.38	0.27
TN09	PLACEBO	CTLA4_PL_Pt9057818	Male	16.30	16.53	0.23	0.30	0.77	43.4	7	61.70	177.00	-0.47	32	2	high	G3	PL_7Fast	1.05	0.70	0.34	0.11	0.06	0.02
TN09	PLACEBO	CTLA4_PL_Pt9062776	Male	8.24	8.49	0.25	0.28	0.18	46.5	7.5	28.40	129.30	0.54	71	3	high	G3	PL_7Fast	0.27	0.10	0.11	0.07	0.03	0.01
TN09	PLACEBO	CTLA4_PL_Pt9080548	Female	17.68	17.93	0.25	0.18	1.76	37.2	6	69.50	167.30	0.89	81	4	high	G3	PL_7Fast	1.17	0.97	0.76	0.43	0.39	0.15
TN09	PLACEBO	CTLA4_PL_Pt9092781	Male	34.06	34.27	0.21	-0.10	3.72	30.4	4.9	94.25	183.70	0.41	66	3	high	G2	0	0.87	1.18	1.00	1.24	1.03	0.44
TN09	PLACEBO	CTLA4_PL_Pt9093522	Male	14.06	14.33	0.27	0.18	4.25	36.0	5.8	63.70	161.30	1.35	91	1	high	G4	0	1.39	1.16	1.07	1.01	0.68	0.73
TN09	PLACEBO	CTLA4_PL_Pt9096907	Male	15.17	15.44	0.27	0.23	2.25	44.6	7.2	77.30	174.50	1.36	91	3	high	G4	0	1.14	0.58	0.42	0.67	0.38	0.32
TN09	PLACEBO	CTLA4_PL_Pt9098761	Male	9.97	10.19	0.22	0.09	1.77	40.9	6.6	35.20	143.50	0.02	57	3	high	G3	0	0.59	0.52	0.45	0.39	0.34	0.17
Mean TN09 Subset (n=74)				14.71	14.99	0.24				6.34	55.04	154.82	0.46	64.23	2.69				0.80	0.71	0.66	0.59	0.47	0.44
SD TN09 Subset (n=74)				6.81	6.73	0.04				0.84	21.52	26.24	0.93	27.50	0.91				0.42	0.40	0.44	0.46	0.42	0.49
Mean TN09 Whole Set (n=112)				14.19	14.43	0.24				6.45	53.04	154.04	0.45	64.05	2.76				0.75	0.65	0.60	0.52	0.44	0.40
SD TN09 Whole Set (n=112)				6.40	6.41	0.04				0.87	21.12	23.82	0.87	25.96	0.89				0.39	0.38	0.42	0.43	0.39	0.44
p-value (2 sample ttest)				0.60	0.57	0.90				0.41	0.53	0.84	0.98	0.96	0.60				0.45	0.32	0.40	0.33	0.60	0.53

ESM TABLE 1 Subject Information LOCAL

Study	Sample Identifier	Gender	Age at diagnosis	Age at visit	Duration of DM @ visit	I.I.359	Length of Partial Remission years IDAA1C > 9	HbA1C at Diagnosis (mmol/mol)	HbA1C at Diagnosis (%)	Weight (kg)	Height (cm)	BMI SDS	BMI Percentile	Number of Detected Autoantibodies	Used for: Treg (T) enumeration or CTLA4Ig Addback (A)	HLA Risk (High: DR3 and/ or DR4 positive; Low nonDR3 and nonDR4)
LOCAL	RO2350	Female	7.25	7.56	0.3123	NA	0.33	72.5	11.7	25.9	126.1	0.35	64	4	T	high
LOCAL	RO2527	Male	10.97	11.29	0.326	NA	0.86	57.7	9.3	39.7	148	0.32	63	4	T	high
LOCAL	RO2973	Female	13.92	14.05	0.1342	NA	1.1	86.8	>14	70.1	161.8	1.58	94	4	T	high
LOCAL	RO3291	Female	6.35	6.83	0.4767	NA	0.28	71.9	11.6	31.2	134.9	0.89	81	4	T	high
LOCAL	RO3325	Male	9.66	10.12	0.4575	NA	1.01	78.7	12.7	43.5	145.9	1.29	90	4	T	high
LOCAL	RO3373	Female	6.79	7.17	0.3808	NA	0.29	86.8	>14	25.9	129.3	-0.01	50	0	T	high
LOCAL	RO3593	Female	11.44	11.56	0.1233	NA	0.74	85.6	13.8	34.7	148.5	-0.95	17	2	T	NA
LOCAL	RO3664	Female	15.73	16.08	0.3534	NA	0.4	NA	NA	53.6	151.1	0.79	79	1	T	low
LOCAL	RO3676	Male	11.45	11.78	0.3342	NA	0.82	64.5	10.4	80	172.8	1.99	98	4	T	high
LOCAL	RO3757	Male	5.64	6.08	0.4438	NA	0.74	42.2	6.8	26.6	124.4	1.11	87	2	T	low
LOCAL	RO3775	Female	16.97	17.14	0.1726	NA	1.1	54.6	8.8	65.6	169.3	0.55	71	3	T	low
LOCAL	RO3803	Female	10.40	10.85	0.4548	NA	0.87	86.8	>14	36.6	148.5	-0.33	37	3	T	low
LOCAL	RO3810	Female	16.65	17.06	0.4082	NA	1.1	81.2	13.1	57.1	172	-0.61	27	4	T	low
LOCAL	RO3887	Male	7.27	7.34	0.0712	NA	0.3	52.7	8.5	24.8	127	-0.15	44	4	T	NA
LOCAL	RO3940	Female	10.82	11.15	0.3205	NA	0.34	86.8	>14	41.5	142	0.93	82	4	T	NA
LOCAL	RO253	Male	10.38	10.74	0.3616	0.73	0.53	49.6	8	60.2	157.5	1.83	97	4	NA	low
LOCAL	RO946	Female	10.68	10.84	0.1562	-0.05	0.3	74.4	12	49.2	148.5	1.37	91	4	NA	high
LOCAL	RO949	Male	9.81	10.34	0.5315	0.40	0.53	71.3	11.5	38.7	133.5	1.5	93	0	T	high
LOCAL	RO972	Male	11.47	11.75	0.2822	0.36	0.28	77.5	12.5	38.5	142.5	0.5	69	4	NA	low
LOCAL	RO1123	Female	13.12	13.34	0.2219	0.41	0.95	73.2	11.8	54	166	0.22	59	0	NA	low
LOCAL	RO1964	Female	9.75	9.84	0.0849	0.71	0.34	69.4	11.2	43.6	155.5	0.5	69	4	NA	high
LOCAL	RO1997	Male	12.75	12.98	0.2274	0.14	0.8	86.8	>14	49.8	153.2	0.88	81	3	T	high
LOCAL	RO2034	Male	5.82	5.96	0.137	0.43	0.83	49.0	7.9	25.2	126.2	0.32	63	4	NA	low
LOCAL	RO2130	Male	15.39	15.62	0.2274	0.77	0.32	68.8	11.1	82	188	0.88	81	2	NA	low
LOCAL	RO2348	Female	9.38	9.65	0.2685	0.71	0.55	67.6	10.9	27.3	136.8	-1.16	12	4	NA	low
LOCAL	RO2359	Female	11.88	12.04	0.1589	0.01	2.41	86.8	>14	45.1	160.7	-0.25	40	3	NA	high
LOCAL	RO2559	Male	11.39	11.8	0.4055	0.52	1.08	72.5	11.7	41.6	150	0.31	59	4	NA	high
LOCAL	RO2808	Male	9.39	9.55	0.1644	0.75	0.59	86.8	>14	48.5	145.1	1.87	97	4	A	high
LOCAL	RO2936	Male	11.55	11.85	0.2959	0.12	1.34	56.4	9.1	31.5	134	-0.07	47	3	NA	high
LOCAL	RO2944	Female	6.36	6.49	0.1315	0.76	0.28	>86.8	>14	25.4	127.2	0.24	59	4	NA	low
LOCAL	RO2953	Female	8.92	9.12	0.2027	1.27	0.29	52.7	8.5	30.6	135.2	0.18	57	3	A	low
LOCAL	RO3052	Female	8.38	8.98	0.6027	0.10	1.86	78.1	12.6	39	145	0.87	81	3	A	high
LOCAL	RO3139	Female	12.03	12.47	0.4329	0.01	0.89	69.4	11.2	40.8	150.7	-0.17	43	4	T	high
LOCAL	RO3309	Female	6.91	7	0.0904	0.70	0.29	56.4	9.1	19.5	119.1	-1.32	9	1	T	high
LOCAL	RO3367	Male	5.19	5.48	0.2904	0.90	0.56	53.9	8.7	18.5	114	-1.1	14	2	T	high
LOCAL	RO3521	Male	5.91	6.13	0.2137	0.31	0.84	57.7	9.3	21.3	114	0.68	75	3	T	high
LOCAL	RO3527	Female	4.47	4.86	0.3836	-0.02	1.23	49.0	7.9	18	109.2	-0.05	48	4	T	high
LOCAL	RO3555	Male	10.68	10.9	0.2219	0.46	0.29	67.6	10.9	56.9	156	1.68	95	3	A	low
LOCAL	RO3572	Male	4.88	5.16	0.2767	0.62	0.63	56.4	9.1	20.6	112.4	0.69	75	4	T	high
LOCAL	RO3585	Male	5.78	6.26	0.474	0.27	0.5	76.3	12.3	23.6	125.9	-0.42	34	4	NA	high
LOCAL	RO3621	Female	8.61	8.91	0.2932	0.52	0.34	78.1	12.6	28.2	128.6	0.35	64	3	NA	low
LOCAL	RO2598	Male	6.21	6.71	0.4959	0.54	0	67.6	10.9	22.1	113.8	0.95	83	4	A	high
Mean TN09 Subset (n=74)			14.71	14.99	0.24					55.04	154.82	0.46	64.23	2.69		
SD TN09 Subset (n=74)			6.81	6.73	0.04					21.52	26.24	0.93	27.5	0.91		
Mean Local (n=42)			9.72	10.02	0.3					39.44	141.67	0.45	63.79	3.14		
SD Local (n=42)			3.27	3.26	0.14					16.45	18.57	0.83	25.23	1.22		
p-value (2 sample ttest)			2.05E-05	1.68E-05	1.14E-03					8.79E-05	5.00E-03	0.98	0.93	0.02		

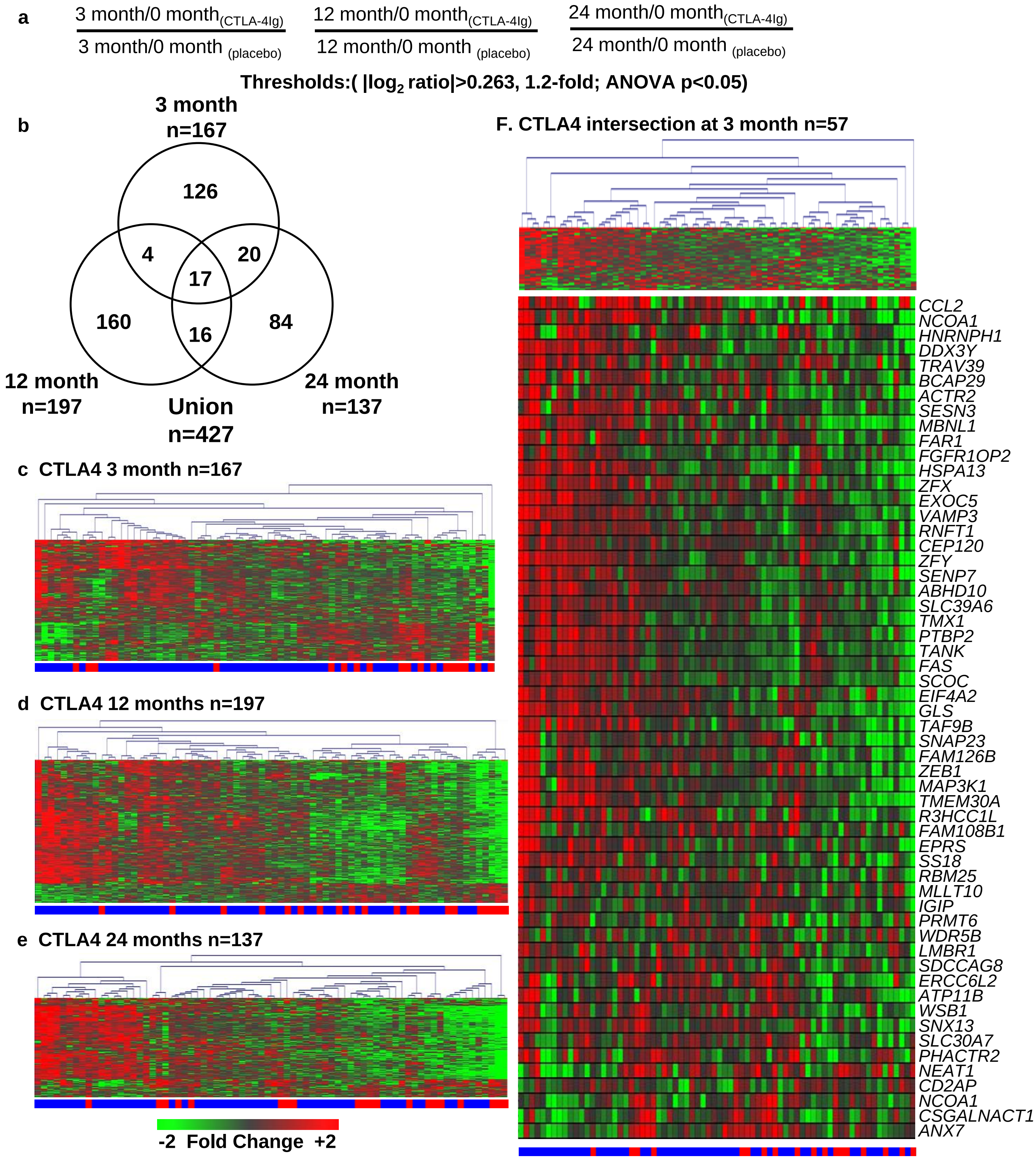

ESM Figure 1. This analysis of the TN-09 trial included 54 CTLA4-Ig-treated patients and 20 placebo-treated patients. Plasma induced transcription was conducted at the baseline, 3 month, 12 month, and 24 month time points. **(a)** Analysis strategy. For each subject, gene expression measured at 3, 12, and 24 months was baseline normalized. **(b)** Venn diagram illustrating the total of 427 probe sets that were differentially induced ($|\log_2 \text{ratio}|>0.263$, 1.2-fold; ANOVA $p<0.05$) between the treatment and placebo arms at ≥1 time point. **(c-e)** Two-way hierarchical clustering (probe sets and subjects) for the regulated probe sets respectively identified between CTLA4-Ig-treated (blue bar) and placebo-treated (red bar) subjects at 3 (n=167 probe sets), 12 (n=197 probe sets), and 24 months (n=137 probe sets) was conducted. There was heterogeneity in both study arms, as evidenced the imperfect clustering observed at each time point. **(f)** An intersection of 57 probe sets, regulated between the two arms at all time points was identified. Well annotated transcripts of the intersection are shown.
